# Intersecting Paths: Unraveling the Complex Journey of Cancer to Bone Metastasis

**DOI:** 10.3390/biomedicines12051075

**Published:** 2024-05-13

**Authors:** Nour Arakil, Shahid Akhtar Akhund, Basant Elaasser, Khalid S. Mohammad

**Affiliations:** Department of Anatomy, College of Medicine, Alfaisal University, Riyadh 1153, Saudi Arabia; narakil@alfaisal.edu (N.A.); sakhund@alfaisal.edu (S.A.A.); belaasser@alfaisal.edu (B.E.)

**Keywords:** bone metastases, bone microenvironment, bone remodeling, interleukin, therapy

## Abstract

The phenomenon of bone metastases presents a significant challenge within the context of advanced cancer treatments, particularly pertaining to breast, prostate, and lung cancers. These metastatic occurrences stem from the dissemination of cancerous cells into the bone, thereby interrupting the equilibrium between osteoblasts and osteoclasts. Such disruption results in skeletal complications, adversely affecting patient morbidity and quality of life. This review discusses the intricate interplay between cancer cells and the bone microenvironment, positing the bone not merely as a passive recipient of metastatic cells but as an active contributor to cancer progression through its distinctive biochemical and cellular makeup. A thorough examination of bone structure and the dynamics of bone remodeling is undertaken, elucidating how metastatic cancer cells exploit these processes. This review explores the genetic and molecular pathways that underpin the onset and development of bone metastases. Particular emphasis is placed on the roles of cytokines and growth factors in facilitating osteoclastogenesis and influencing osteoblast activity. Additionally, this paper offers a meticulous critique of current diagnostic methodologies, ranging from conventional radiography to advanced molecular imaging techniques, and discusses the implications of a nuanced understanding of bone metastasis biology for therapeutic intervention. This includes the development of targeted therapies and strategies for managing bone pain and other skeletal-related events. Moreover, this review underscores the imperative of ongoing research efforts aimed at identifying novel therapeutic targets and refining management approaches for bone metastases. It advocates for a multidisciplinary strategy that integrates advancements in medical oncology and radiology with insights derived from molecular biology and genetics, to enhance prognostic outcomes and the quality of life for patients afflicted by this debilitating condition. In summary, bone metastases constitute a complex issue that demands a comprehensive and informed approach to treatment. This article contributes to the ongoing discourse by consolidating existing knowledge and identifying avenues for future investigation, with the overarching objective of ameliorating patient care in the domain of oncology.

## 1. Introduction

Bone metastases provide a substantial clinical obstacle, predominantly impacting patients with advanced malignancies such as breast, prostate, and lung cancer [1,2]. The metastasis of cancer cells to the bone highlights the significance of the bone microenvironment in the course of cancer. Bone metastases disturb the intricate equilibrium between bone formation and resorption, which is mainly regulated by osteoblasts and osteoclasts. This disruption results in skeletal problems that have a substantial impact on patient morbidity and quality of life [3]. Bone metastases arise when cancer cells detach from the initial tumor, disseminate throughout the body, and form secondary tumors inside the bone tissue. This intricate process entails interactions between cancer cells and the bone microenvironment, ultimately resulting in the disturbance of regular bone remodeling processes.

The occurrence of bone metastases varies among different cancer types, but it is particularly prevalent in people with advanced breast, prostate, and lung malignancies. Bone metastases have a significant effect on patients, causing intense pain, a higher likelihood of fractures, compression of the spinal cord, and hypercalcemia, underscoring the importance of implementing good management approaches [4]. These consequences not only result in a reduced quality of life but also contribute to higher mortality rates among cancer patients. Comprehending the mechanisms that cause bone metastases is vital in the fields of oncology and musculoskeletal problems. This understanding is essential for the development of precise treatments that can alleviate these effects and enhance patient outcomes.

The significance of bone metastases in the field of oncology and musculoskeletal problems cannot be overemphasized. The bone microenvironment is a critical region for the spread of various types of cancer, serving as a prominent location for metastasis. It plays a vital role in supporting the survival and growth of cancer cells. The interaction between cancer cells and the bone matrix emphasizes the significance of focusing on the bone microenvironment while treating bone metastases.

### 1.1. Basic Structure of Bone

Bones are the complex components that make up the human body’s framework. These allow for mobility, protection, and support. Bone tissue is a dynamic network of cells, proteins, and minerals that is constantly changing during the course of a person’s life [5,6]. This process, which requires a careful balance between osteoblasts’ production of new bone and osteoclasts’ resorption of existing bone, ensures the preservation of bone integrity, strength, and flexibility. Through complex signaling networks, hormonal control, and mechanical loading, bone remodeling adjusts to a range of physiological needs, including growth, repair, and stress responses to the environment. It is important to understand how molecular mechanisms and cellular activities affect bone remodeling and structure in order to better understand bone physiology and pathophysiology. This, in turn, leads to better bone health and therapeutic interventions. Bone is a sophisticated and structurally complicated biomaterial. Four different cell types, which are osteoblasts, osteocytes, osteoclasts, and bone lining cells, are found in bone and are responsible for the main remodeling function [5,7].

#### 1.1.1. Osteoblasts

The osteoblast is the primary cell in charge of bone development [8]. After passing through various stages of development, the mesenchymal stem cells (MSCs) found in bone marrow give rise to osteoblasts under the influence of various molecular factors, which mainly belong to bone morphogenic proteins (BMPs) [9]. Osteoblasts are mononucleate, cuboidal cells [10]. The ultrastructural features of osteoblasts include abundant cytoplasm containing the rough endoplasmic reticulum [11]. Osteoblasts are polarized and align themselves linearly on the formed bone. These cells make osteoid: a precursor bone. Osteoblasts secrete alkaline phosphatase, which is believed to be involved in osteoid mineralization. The level of this enzyme rises during the process of forming new bone during tumor growth and fracture, as well. It serves as a clinical indicator of bone activity. In the process of making bones, osteoblastic cells may also circulate in serum [12]. Osteoblasts are mechanically sensitive and can translate mechanical pressures into biochemical signals that affect cellular activity and gene expression. According to research, osteoblasts react to situations involving both mechanical loading and unloading [8]. Osteoblast function dysregulation is a factor in bone disorders.

#### 1.1.2. Osteocytes

Osteocytes represent most bone cells. These are found embedded within the mineralized bone matrix [13]. They connect to surface bone cells and to one another through cytoplasmic processes that are thin and radiate in different directions, usually perpendicular to the surface of the bone. These processes move through canaliculi, which are microscopic channels found in bone that allow osteocytes and surface cells to communicate. Osteocytes are essential to the development and function of bones, and their initial characteristic was that they were found embedded within bone tissue [14]. Osteocytes were previously identified primarily by their location within bone tissue, but now these are also known for their multifunctional roles in bone remodeling and other metabolic functions. They serve as endocrine cells, influencing the activities of osteoblasts, osteoclasts, and other organs. Additionally, they play crucial roles in maintaining phosphate and calcium balance, exerting targeted effects on the kidney and participating in perilacunar remodeling. Furthermore, osteocytes act as mechanosensory cells within the skeleton, earning them the title of “master regulators” of bone [13,14].

#### 1.1.3. Osteoclast

Osteoclasts are multinucleated cells with a unique ability to resorb bone. These are distinguished by unique characteristics, including a ruffled border, which promotes bone matrix deterioration and demineralization [15]. During the embryonic and neonatal stages, erythroid-myeloid progenitors (EMPs) emerge around embryonic day 7 within the yolk sac’s blood island. These EMPs have the capacity to differentiate into colony-stimulating factor 1 receptor (Csf1r)+ yolk sac macrophages, acting as precursors for osteoclasts [16]. In postnatal life, these cells are replaced with hematopoietic stem cells and differentiate through a multistep process influenced by various factors, including M-CSF and RANKL [17]. Therapeutic interventions often target osteoclasts to combat bone loss. Traditionally, scientists viewed them as having a linear life cycle. However, recent research using advanced techniques has revealed their extended lifespan, ability to circulate, and ability to undergo cell recycling. Contrary to previous beliefs, under normal conditions, osteoclast apoptosis is rare, challenging previous understandings of their turnover dynamics [18].

### 1.2. Bone Remodeling

As a dynamic tissue, bone is constantly remodeling itself [19]. This process takes place inside the bone cavities [5]. Osteoblasts and osteoclasts mostly carry out the process of remodeling, and different molecular mechanisms regulate this process. Together, these cells create transitory structures called basic multicellular units [5], which are to rebuild bone [20]. The basic multicellular unit (BMU) is where this communication takes place during the beginning, middle, and end stages of bone remodeling [21]. Specific cells must coordinate bone resorption and production for proper bone development and mature skeletal adaptability [7]. Direct cell-to-cell contact, diffusible paracrine substances, and interactions with the bone matrix are the three main ways that cells within the osteoclast and osteoblast lineages interact [21]. Osteoblasts initiate and mineralize new bone and control the processes of osteoclastogenesis and bone resorption. Osteoblasts are essential to the growth and remodeling of the skeleton. There is evidence that cancer cells also use osteoblasts as a means of colonizing bone and promoting the formation of tumors. Besides osteoclasts and osteoblasts, osteocytes are also suggested to be orchestrators of bone remodeling [22].

**Figure 1:**
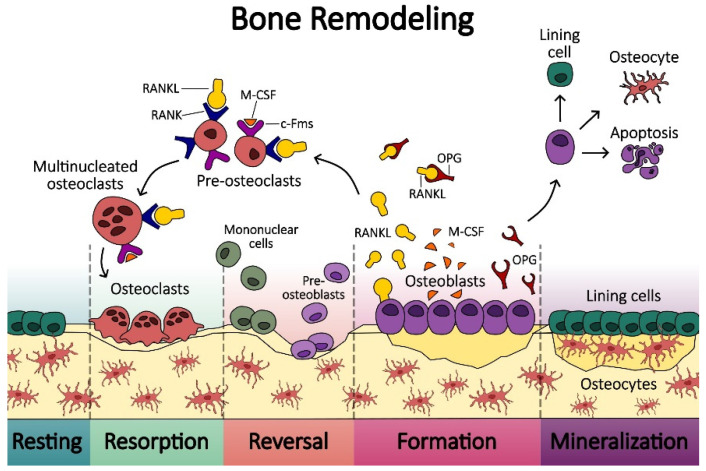
The figure illustrates the complex cycle of bone remodeling, divided into five phases: resorption, reversal, formation, resting, and mineralization. At the outset, RANKL (Receptor Activator for Nuclear Factor κ B Ligand) binds to its receptor, RANK, on pre-osteoclasts, in the presence of M-CSF (macrophage colony-stimulating factor), promoting the maturation of pre-osteoclasts into multinucleated osteoclasts capable of resorbing bone. Osteoprotegerin (OPG) acts as a decoy receptor for RANKL, inhibiting osteoclastogenesis and thus bone resorption. During the resorption phase, osteoclasts break down bone tissue, releasing calcium and phosphorus into the blood. Following resorption, the reversal phase begins, characterized by the transition from osteoclast activity to osteoblast activity. Mononuclear cells appear, which may play a role in recruiting osteoblast precursors to the remodeling site. The formation phase sees the recruitment of pre-osteoblasts, which differentiate into osteoblasts. These osteoblasts synthesize new bone matrix in the resorbed area, which is then mineralized by the deposition of calcium and phosphorus into the bone matrix to form new bone tissue. Osteoblasts that become embedded in the bone matrix differentiate into osteocytes, which are critical for bone metabolism and signaling. Some osteoblasts remain on the bone surface as lining cells, which protect the bone and regulate the passage of calcium. Finally, the bone enters a resting phase before the remodeling cycle recommences. This cyclical process ensures the renewal of bone tissue, maintaining bone strength and mineral homeostasis in the body.

During the bone remodeling process, in the BMU, a group of osteoblasts forms the closing cone, and a group of osteoclasts forms the cutting cone. It has been proposed that the bone remodeling compartment (BRC), which surrounds the BMU, is made up of a canopy of cells that may include bone lining cells. The BRC appears to be associated with the cells that line the surface of bones, which are in contact with the osteocytes that are encased in the bone matrix [5,23]. The bone remodeling process has various phases [5]. The initial phase is bone resorption, followed by bone formation. Between the two phases is the period of transition or reversal, and following the formation phase a mineralization phase starts (Figure 1). There is spatial and temporal coordination between these processes. This process is referred to as coupling [24]. In this process, osteoclasts and many coupling factors released by osteoclasts play a central role in the commination between bone cells involved in remodeling.

Numerous factors, such as hormones, cytokines, and mechanical forces, affect bone turnover, which affects the amount and caliber of new bone tissue that is formed. The initial phase is under the influence of RANKL and M-CSF [25]. Mechanical stress is very important for maintaining bone mass and preventing bone loss associated with aging. Even with cumulative loading damage, bone can mend itself; the molecular principles underlying this ability are becoming increasingly clear. The health of bones, illnesses, and adaptability to weightless surroundings are all significantly impacted by these processes [20].

## 2. Bone Metastases and Tumor–Bone Interaction

**Figure 2:**
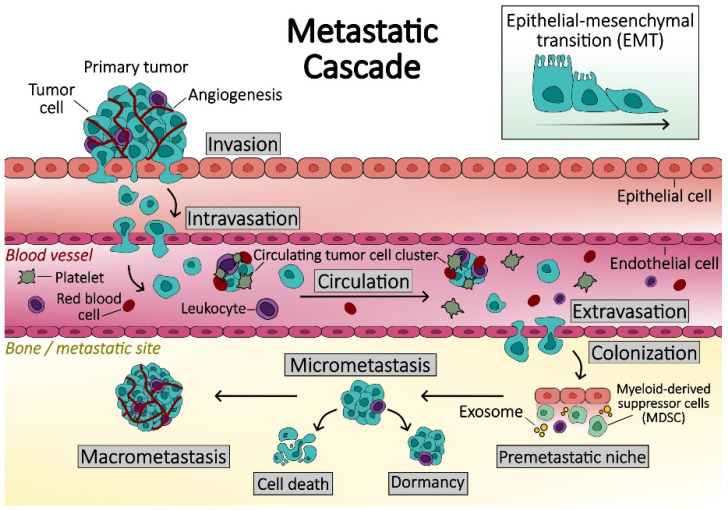
This figure illustrates the complex process of cancer metastasis. The metastatic process in cancer is a multi-stage event that involves the transformation and movement of cancer cells from the primary tumor site to secondary locations in the body. The metastatic cascade begins with the tumor growth and angiogenesis, where new blood vessels form to supply nutrients to the tumor. Cancer cells then undergo epithelial–mesenchymal transition (EMT), a critical change that enhances their mobility and invasiveness, allowing them to breach the extracellular matrix and invade nearby tissues. The next phase, intravasation, sees these cells entering the bloodstream, where they can circulate as individual cells or clusters, interacting with blood components such as platelets and leukocytes. Upon reaching a distant site, the circulating tumor cells exit the bloodstream through extravasation. In the new tissue environment, these cells can remain dormant or progress to colonize, recruiting supporting cells such as myeloid-derived suppressor cells (MDSCs) and creating a premetastatic niche that facilitates the growth of micrometastasis into a full-blown macrometastasis. Throughout this journey, the interplay between cancer cells, the host’s immune system, and the microenvironment of both the primary and metastatic sites are crucial for the successful establishment and growth of metastases.

The development of bone metastases is a sequential process initiated when tumor cells detach from the primary site and invade adjacent tissues. These cells enter the bloodstream through a mechanism termed intravasation, subsequently circulating as circulating tumor cells (CTCs) capable of disseminating to distant sites, including bones and other organs [26]. Upon extravasation and migration to premetastatic niches, they become disseminated cancer cells (DCCs), localized within specific microenvironments, notably the vascular and osteoblastic or endosteal niches, which are critical for their survival [27]. DCCs may either undergo apoptosis or survive and form micrometastases or enter a dormant state. Dormant tumor cells may proliferate into a micrometastatic cluster, through poorly understood processes, and then adapt to the bone microenvironment, in a phase known as colonization [28]. Tumor-derived products induce osteolysis, releasing bone-derived factors that support tumor cell growth, perpetuating a self-sustaining cycle of bone degradation known as the vicious cycle of bone metastasis. Ultimately, tumor cells grow into macroscopic lesions, disrupting the delicate balance between bone resorption and formation (Figure 2).

Numerous factors have been identified that facilitate the propensity of malignant cells to metastasize to bone, including the intrinsic properties of the tumor cells, the unique attributes of the bone microenvironment, and the dynamic interactions between them. This bidirectional communication between tumor cells and the bone microenvironment fosters both osteolysis and the proliferation of metastases. The bone microenvironment, comprising the mineralized extracellular matrix and diverse cellular inhabitants, serves as a conducive “soil” for the implantation and proliferation of metastatic cancer “seeds [29]”.

It is critical to acknowledge that a mere fraction of disseminated cancer cells (DCCs) or micrometastases possess the capacity to evolve into mature metastatic lesions, attributed to the inherent inefficiency of the metastatic cascade. Indeed, only a nominal proportion of about 0.02% of DCCs advance to form detectable metastases. The majority of DCCs encounter substantial challenges within the dynamic and hostile microenvironment during metastasis [30]. Analysis of bone marrow micrometastases in breast cancer revealed that the incidence of DCCs in the bone marrow surpasses the prevalence of patients developing skeletal metastases. Nevertheless, the detection of DCCs in bone marrow is prognostic of the potential for skeletal metastasis development [31,32].

Entry into a dormant state enables DCCs to endure prolonged periods, eluding immune surveillance through mechanisms presently under investigation. Furthermore, dormancy confers resistance against conventional cancer therapies targeting rapidly proliferating cells. Post-therapy, dormancy facilitates the persistence of tumor cells as minimal residual disease (MRD), thereby elevating the risk of cancer recurrence [33]. Particularly after the eradication of a primary tumor, there is a heightened likelihood of relapse in cases such as breast cancer metastases, with parallel observations in prostate cancer metastases and other cancer types.

## 3. Disruption of Osteoclast–Osteoblast Balance by Metastatic Cells

Tumor metastasis is a complicated and poorly understood process that involves various steps. To separate from the parent tumor and reach the lymphatic or circulatory systems, the cancer cells go through epithelial-to-mesenchymal transition (EMT) [34]. Subsequently, these circulating tumor cells (CTCs) may travel to other organs and become disseminated tumor cells (DTCs), which settle in a secondary microenvironment [35]. Bone marrow is the site of choice for DTCs because it has an abundance of growth factors, neovascularization factors, cytokines, and chemokines [36]. However, the bone microenvironment presents a challenge for the tumor cells to settle and grow [37]. The growth of tumor cells within the bone and the process of bone remodeling are considered to be closely related [38]. Any disruption in the dynamic equilibrium of bone formation and resorption results in diseases that provide suitable seats for the tumor cells to grow [39]. Due to the unfavorable microenvironment of distant organs, the DTCs stay in dormancy [40]. According to Croucher [37] and Haydar [41], for cancer cells to progress in the bone, they undergo four phases: (a) colonization of bone marrow with cancer cells; (b) dormancy, when cancer cells do not proliferate for a long time and adapt to the bone; (c) reactivation and development, when cancer cells come out of dormancy and begin to proliferate actively; and (d) reconstruction, when cancer cells change the original structure and function of the bone.

It is proposed that even before the tumor cells even reach the bone for colonization, they help create a premetastatic niche in the bone [35]. Various factors like interleukin (IL)-6, tumor-derived IL-1β; Hypoxia-induced lysyl oxidase (LOX) secreted by the primary tumor cells; and cancer cell-derived exosomes like DNA, RNA [mRNA, microRNA (miRNA), and other noncoding RNAs], lipids, and proteins released by all types of cells and taken up by recipient cells are involved in the formation of a premetastatic niche in the bone [35].

Osteoclasts and osteoblasts either directly interact with dormant tumor cells or secrete substances that control tumor cells’ dormant condition in close proximity to the surface of the bone. On the other hand, to come out of dormancy and become active, dormant tumor cells may attract osteoclast progenitors and increase local osteoclast activity. This implies that dormant tumor cells may also have an impact on the control of the “on-and-off dormancy switch” in osteoblasts and osteoclasts [42,43].

It is not uncommon to see those metastatic cells in the bone result in an imbalance between bone formation and bone resorption. This is because of overactivation of either osteoblast or osteoclast cells, respectively [34]. On the one hand, tumor cells release various factors that promote osteoclast activity and bone resorption, including the parathyroid hormone-related protein (PTHrP), lysophosphatidic acid (LPA), macrophage-stimulating protein (MSP), prostaglandin E2 (PGE2), interleukin-8 (IL-8), interleukin-11 (IL-11), matrix metalloproteinase-1 (MMP-1), CCN3, platelet-derived lysophosphatidic acid (LPA), and granulocyte–macrophage colony-stimulating factor [44,45,46,47,48]. On the other hand, tumor cells not only enhance osteoclast activity but also suppress osteoblast function, exacerbating the imbalance between bone formation and resorption, ultimately leading to bone destruction [34,49].

Activin A, the BMP inhibitor noggin, dickkopf-1 (DKK-1), and sclerostin (SOST-1) are mainly responsible for osteoblast suppression [50,51,52,53,54,55]. Contrary to osteoblastic suppression, some tumors secrete factors that can promote osteoblastic activity, hence osteosclerotic bone lesion [56]. In the osteosclerosis process, among various factors that stimulate osteoblast proliferation and inhibit osteoclast activity and motility, endothelin-1 (ET-1) was recognized as a major mediator [57]. Tumor cells that trigger osteoblastic lesions can both stimulate osteoblast activity and, occasionally, suppress osteoclast activity. Cancer cells produce osteoclast inhibitors such as endothelin-1 (ET-1) and osteoprotegerin (OPG) [58].

### 3.1. The Role of Adipocytes in the Bone Microenvironment

Bone marrow adipocytes (BMAs) are abundant adipose cells residing within the bone marrow and that arise from bone marrow mesenchymal stromal cells [59]. The contribution of BMAs to the tumor–bone microenvironment is both tumor-promoting and tumor-suppressing. While many studies have explored the link between BMAs and bone metastasis, the direct mechanisms through which BMAs lead to bone metastasis development and progression remain unclear.

**Figure 3:**
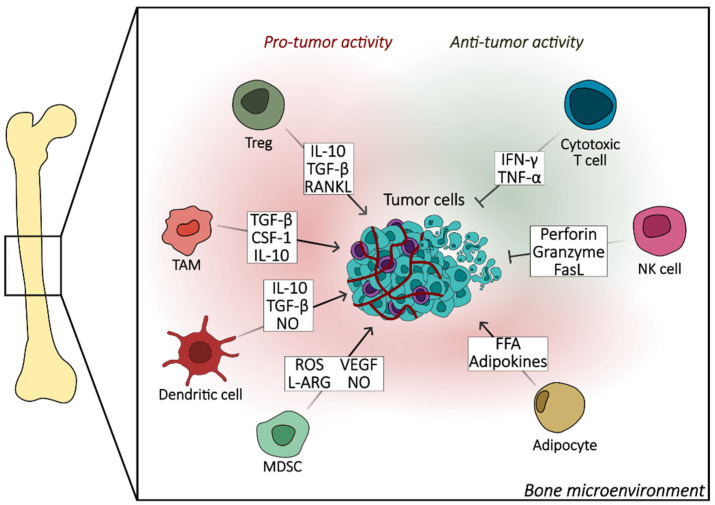
Interactions in the bone microenvironment influencing tumor activity. This figure illustrates the dual roles of various immune cells and factors within the bone microenvironment, delineating their pro-tumor and anti-tumor activities. Key components include cytotoxic T cells, natural killer (NK) cells, regulatory T cells (Tregs), myeloid-derived suppressor cells (MDSCs), dendritic cells, adipocytes, and tumor cells. The figure highlights the secretion of cytokines such as interleukin-10 (IL-10), transforming growth factor-beta (TGF-β), and tumor necrosis factor-alpha (TNF-α), along with other factors like reactive oxygen species (ROS), nitric oxide (NO), and various adipokines, showcasing their impact on tumor cell survival and proliferation within the bone microenvironment.

BMAs secrete various cytokines, growth factors, and adipokines (Figure 3) that influence the behavior of tumor cells and the microenvironment [60]. Two adipocyte-derived inflammatory cytokines, interleukin-6 (IL-6) and tumor necrosis factor-alpha (TNF-α), promote the development of bone metastasis. IL-6 has been shown to induce epithelial–mesenchymal transition (EMT) promoting metastasis [61]. Moreover, BMAs influence the process of bone formation by modulating the activity of osteoblasts and osteoclasts. BMAs can also stimulate osteoclastogenesis through RANKL expression [62].

Adipokines, such as leptin, adiponectin, and adipsin, activate several signaling cascades that have been implicated in metastasis such as angiogenesis, proliferation, and cell migration [63,64,65]. Leptin, a pro-inflammatory adipokine, serves an important role in energy homeostasis and bone density modulation. When leptin binds to central receptors, it promotes bone loss and when it binds to peripheral receptors, it promotes bone gain [66]. Leptin plays a pro-tumor role, and on the other hand, adiponectin, an anti-inflammatory adipokine, has been shown to exert an anti-tumor effect [67,68]. Despite growing evidence supporting adiponectin’s anti-metastatic effects, studies are reporting its role in the promotion of tumor progression and metastasis [69,70]. Another notable characteristic of BMAs is their ability to contribute energy in the form of free fatty acids to tumor cells in bone metastasis [71].

### 3.2. Immune System and Bone Metastases

The modulation of immune responses plays a pivotal role in the progression and control of metastatic cancer. Treg cells play a crucial role in the immune response against bone metastases. They enter tumor tissues and facilitate immunosuppression, which obstructs the body’s ability to fight against the tumor [72]. Within the bone microenvironment, a multitude of immune cells, including Tregs, interact with cancer cells, influencing metastasis [73]. Targeting Tregs in cancer therapy shows promise, as inhibiting Tregs can enhance the response to radiotherapy and improve control of disease progression. Specific elimination of tumor antigen-specific Tregs, especially those infiltrating breast tumors, has been shown to restore antitumor immune responses and enhance the efficacy of immune checkpoint inhibitors [74]. Strategies such as mAb-mediated depletion and functional blockade of Tregs are being explored for their potential in developing efficient and safe combinatorial immunotherapies for bone metastases [75].

The activation of myeloid-derived suppressor cells (MDSCs) plays a significant role in the progression of bone metastases. Studies have shown that factors like Galectin-1 (Gal1) and beta 2 adrenergic receptor (β2AR) activation can lead to the expansion and activation of MDSCs in the pre-metastatic bone microenvironment. These activated MDSCs contribute to the establishment of a pro-tumoral niche by altering the local microenvironment to support metastatic spread through mechanisms such as collagen and extracellular matrix remodeling [76]. Additionally, MDSCs can counterbalance anti-tumoral immunity by suppressing cytotoxic T cells, promoting tumor growth in the bone cavity [77]. The activation of MDSCs via the IL6-STAT3-Arg1/IDO1 pathway creates a conducive environment for bone metastasis progression.

The activation of cytotoxic T cells in bone metastases leads to the release of TNF alpha and INF gamma through a coordinated migration process resembling neutrophil swarming. This migration involves the acceleration of distant T cell recruitment via long-range homotypic signaling, facilitated by chemokines like CCL3 and CCL4 [78]. The newly arriving CTLs amplify the chemotactic signal, creating a positive feedback loop that accelerates mass recruitment of T cells [79]. This intra-population signaling mechanism is also observed in activated effector human T cells and CAR T cells, driving rapid convergence towards the target site. Therefore, the activation of cytotoxic T cells in bone metastases triggers a localized mass response by enhancing the recruitment of additional T cells independently of other leukocytes, ultimately leading to the release of TNF alpha and INF gamma.

Modulating the activity of dendritic cells through TGFβ signaling pathways is crucial in bone metastases [80]. TGFβ promotes tumor cell secretion of factors that accelerate bone loss and fuel tumor cells to colonize bone, driving a vicious cycle of tumor growth [81]. Additionally, TGFβ inhibits the migration of dendritic cells from tumors to draining lymph nodes, increasing the likelihood of metastasis in affected nodes [82]. Furthermore, TGFβ and IL-10 are involved in suppressing dendritic cell function, down-regulating NF-κB activation, and inhibiting the production of key cytokines and chemokines by dendritic cells. Therefore, targeting TGFβ and IL-10 signaling pathways could disrupt the immunosuppressive effects, potentially reverting epithelial–mesenchymal transition, enhancing immune responses, and ultimately impacting the progression of bone metastases.

### 3.3. Role of Cytokines and Growth Factors in Bone Remodeling and Metastases

#### 3.3.1. Role in Bone Remodeling

Understanding the intricate interplay between cytokines, growth factors, and bone metastases is crucial for elucidating the mechanisms underlying the pathogenesis of skeletal complications in cancer [83,84]. Interleukins, initially identified as mediators of immune responses, have emerged as key regulators of bone homeostasis, orchestrating a plethora of cellular processes essential for bone remodeling and metastatic progression. Cytokines, including tumor necrosis factor-alpha (TNF-α) and interleukin-1 (IL-1), exert profound effects on the bone microenvironment by modulating the activity of osteoblasts, osteoclasts, and immune cells. Additionally, a complex network of signaling molecules, such as receptor activator of nuclear factor kappa-B ligand (RANKL) and osteoprotegerin (OPG), governs osteoclastogenesis and bone resorption. Furthermore, a myriad of interleukins, including IL-11, IL-6, and IL-17, along with growth factors like transforming growth factor-beta (TGFβ), intricately regulate osteoclast formation and function. Importantly, an understanding of the balance between pro-osteoclastogenic and anti-osteoclastogenic cytokines, such as interferons and interleukins, provides insights into potential therapeutic strategies for mitigating bone metastases. In this section, we delve into the multifaceted roles of cytokines and growth factors in bone metastases, highlighting their significance as potential targets for therapeutic intervention.

Scientists first thought that leukocytes alone expressed interleukins (ILs), but later discovered that many other body cells also produce them. They play essential roles in immune cell activation and differentiation, as well as proliferation, maturation, adhesion, and migration. They also have pro-inflammatory and anti-inflammatory properties. The primary function of interleukins is to modulate growth, differentiation, and activation during inflammatory and immune responses. Interleukins comprise a large group of proteins that can elicit many reactions in cells and tissues by binding to high-affinity receptors on cell surfaces. They have both paracrine and autocrine function. Animal studies also utilize interleukins to investigate aspects related to clinical medicine. Cytokines play critical roles in regulating host responses to infection, immune reactions, inflammation, and injury. Some cytokines exacerbate diseases by promoting inflammation (pro-inflammatory), others aid in reducing inflammation and fostering healing (anti-inflammatory). Pro-inflammatory cytokines like interleukin-1 (IL-1) and tumor necrosis factor (TNF) can worsen disease conditions when administered to humans [85]. Bone remodeling is governed by a carefully regulated equilibrium among bone cell populations, including osteoblasts responsible for bone formation, osteoclasts involved in bone resorption, and osteocytes, which serve as mechanosensory cells [5,86]. Osteoclastic activity is regulated by various osteoclastogenic and anti-osteoclastogenic cytokines [86].

Bone marrow stromal cells and fibroblasts generate IL-11, which primarily targets hematopoietic progenitors and osteoclasts. IL-11 functions include promoting osteoclast formation, increasing colony-stimulating factor levels, raising platelet count in vivo, and inhibiting the production of pro-inflammatory cytokines [87]. The synovial macrophages and T cells produce TNF-α, interleukin (IL)-1, IL-6, and IL-17 which enhance the osteoclastogenic process by acting on osteoblasts to promote RANKL expression [88]. Various other ILs (IL-1, IL-6, IL-7, IL-8, IL-11, IL-15, IL-17, IL-23, IL-34) and transforming growth factor-β are believed to play a role in osteoclastogenic roles [86]. Osteoclastogenesis and osteoclast function are primarily controlled by a family of closely related tumor necrosis factor (TNF) receptor (TNFR)/TNF-like proteins, namely RANKL (receptor activator of nuclear factor kappa-B ligand), RANK (receptor activator of nuclear factor kappa-B), and osteoprotegerin (OPG) [89,90]. RANKL is a type II transmembrane protein produced by osteoblasts, osteocytes, and immune cells. RANK, a receptor encoded by the TNFRSF11A gene located on chromosome 18 (18q21.33), is primarily found in osteoclast precursors, mature osteoclasts, and immune cells [89]. OPG, a receptor encoded by the TNFRSF11B gene situated on chromosome 8 (8q24.12), functions as a soluble decoy receptor lacking a transmembrane structure. It operates by negatively regulating osteoclastogenesis through its binding to RANKL [91]. Alongside RANKL, RANK, and OPG, macrophage colony-stimulating factor (M-CSF) also holds crucial importance in osteoclast formation and bone resorption activity [92]. While many cytokines have osteoclastogenic activity, numerous have anti-osteoclastogenic and anti-resorptive properties [86]. Various cytokines and interferon have anti-osteoclastogenic properties. IL-3 (C11), IL-4 (C12), IL-10 (C 13), IL-12 (C14), IL-18 (C2), IL-27 (C15), and IL-33 (C16) have this effect through various mechanisms [86]. IL-4 diminishes RANKL-triggered osteoclast formation, while IL-10 directly hampers osteoclast formation. IL-12 not only impedes lipopolysaccharide (LPS)-induced osteoclast formation in vivo but also prompts osteoclast apoptosis. Both IL-13 and IL-27 curb RANKL-induced osteoclast formation by STAT1-mediated suppression of c-Fos, whereas IL-33 mitigates RANKL-induced osteoclast formation. These cytokines collaborate to instigate apoptosis of osteoclast precursors and hinder TNF-α-mediated osteoclastogenesis in myeloid cells. TNF-α induces Fas expression, whereas IL-12 and IL-18 prompt FasL expression, culminating in osteoclast precursor apoptosis. Additionally, interferon (IFN)-α, IFN-β, and IFN-γ also impede osteoclastogenesis [86].

#### 3.3.2. Role in Bone Metastases

The proliferation of tumor cells in the bone microenvironment disrupts the delicate balance of bone remodeling by releasing substances such as interleukins (ILs) and parathyroid hormone-related protein (PTHrP), which prompt osteoblasts to increase the synthesis of receptor activator of NF-κB ligand (RANKL). The process of osteoclast precursors developing into active, bone-resorbing osteoclasts is initiated by the binding of RANKL to the receptor RANK on osteoclasts. Enhanced osteoclast formation and heightened bone resorption lead to the release of substances from the deteriorating bone matrix that support tumor development, such as transforming growth factor-β (TGFβ). Due to this process, the bone metastasis cycle begins, emphasizing the important relationship between tumor and bone cells in driving disease progression [93].

In the microenvironment of metastatic bone lesions, both cancer cells and bone cells secrete interleukins (ILs) and express the appropriate receptors. ILs have been identified as essential regulators in this intricate communication network. It is established that tumor cells and bone cells may also create ILs and react to both autocrine and paracrine IL-signaling [94]. The immunomodulatory cytokine interleukins (ILs) are thought to be important modulators of the bone cell–disseminated tumor cell (DTC) interaction. It has been shown that certain ILs work in a pro-osteoclastogenic manner, controlling the development and activity of bone cells [93]. Furthermore, ILs impact the maturation and function of osteoblasts and osteoclasts [95]. Most ILs have pro-osteoclastogenic effects on osteoclast maturation, differentiation, and function, either directly or indirectly [96]. It has been proposed that ILs can especially encourage cancer stem cells (CSCs) to establish colonies once they reach the metastatic location [97].

The production of interleukin-6 (IL-6) in the bone marrow microenvironment is crucial for the advancement of bone metastases. The strong pro-tumorigenic effects of IL-6 come from its role in bone metabolism, tumor cell proliferation and survival, angiogenesis, and inflammatory responses. Numerous signaling pathways are involved in the orchestration of these effects, including the Ras/mitogen-activated protein kinase (MAPK), phosphoinositol-3 kinase (PI3K)–protein kinase B/Akt (PkB/Akt), and Janus kinase/signal transducer and transcription activator (JAK/STAT-3) pathways. IL-6 starts these pathways’ activation, which is further amplified when the soluble IL-6 receptor (sIL-6R) is present. The association between high serum levels of IL-6 and sIL-6R in patients with bone metastases is noteworthy and suggests a dismal clinical outcome [98]. It is important for the distant tumor cells to leave the blood vessels in order to be seeded in the secondary organs. It has been demonstrated that IL-1ß and IL-6 raise focal adhesion kinase (FAK) by which DTCs engage with endothelial and stromal cells through adhesion molecules for their extravasation from circulation into distant organs [99]. Similarly, tissue-resident iNKT-cell-derived IL-22 activates endothelial cells and induces the expression of ANPEP, thereby promoting extravasation during metastasis formation [100,101].

There has been evidence of a relationship between IL-8 levels and breast CSC activity [102]. IL-8, a chemokine originally identified as a neutrophil chemoattractant, is implicated in the process of osteoclast formation and bone resorption associated with metastatic breast cancer. IL-8 was found to directly stimulate the differentiation of human peripheral blood mononuclear cells into bone-resorbing osteoclasts, even in the presence of excess RANK-Fc, indicating a direct effect on osteoclast differentiation and activity. Human osteoclast precursors and mature osteoclasts express the specific IL-8 receptor CXCR1, suggesting a direct link between IL-8 signaling and osteoclastogenesis. These findings demonstrate IL-8′s involvement in osteolysis associated with metastatic breast cancer and suggest it as a potent activator of bone destruction common in metastatic bone disease [95]. While certain ILs like IL6 and IL8 show a role in CSC presence, the role of ILs related to circulating tumor cells (CTCs) is challenging to establish [98].

Transforming growth factor-beta (TGFβ) family members regulate a variety of biological processes, including cell division, proliferation, migration, and extracellular matrix (ECM) deposition. Bone morphogenetic proteins (BMPs), activins, and TGFβ are among the more than 35 structurally related secreted polypeptides that make up the TGFβ superfamily of growth factors [103]. In mammalian tissues, TGFβ is found in three isoforms: TGFβ1, TGFβ2, and TGFβ3. These isoforms are first produced and released as latent TGFβs (L-TGFβs). Through cleavage or conformational changes in L-TGFβs, TGFβ is activated physiologically. The active TGFβ is formed in acidic cellular conditions or by proteolytic processes that are facilitated by matrix metalloproteinases (MMPs) and other enzymes. After activation, TGFβs bind to their particular signaling receptors, which in turn initiates intracellular signaling cascades through the pathways of mitogen-activated protein kinase (MAPK) and Smad [104]. Recent evidence highlights the crucial involvement of TGFβ signaling in breast cancer metastasis, particularly to the bone. In mouse models, TGFβ facilitates bone metastasis via factors like parathyroid hormone-related peptide, interleukin-11, and CTGF [105]. Studies also underscore Smad signaling necessary for TGFβ-induced bone metastasis. Smad4 depletion can inhibit metastasis, while Smad7 overexpression curbs invasiveness, affirming the role of Smad signaling in breast cancer progression [106].

We have elucidated the crucial functions that cytokines and growth factors serve in the regulation of bone metastasis, highlighting their dual roles in stimulating both osteoclast and osteoblast activity. Upon reflection, it is essential to delve deeper into the therapeutic implications of these discoveries. For instance, despite the validated clinical efficacy of targeting the RANKL/OPG pathway, various cytokines like interleukin-6 and TGFβ, which also exert notable effects on bone homeostasis, could present additional therapeutic opportunities. Considering a clinical standpoint, precision-targeted therapies that regulate these cytokines might potentially improve patient outcomes by finely tuning bone–tumor interactions. Moreover, my examination indicates that forthcoming therapeutic strategies should concentrate on the less investigated cytokines and their receptors, which could introduce innovative pathways to disrupt the metastatic cascade, thereby fostering optimism for interventions aimed at preventing or more efficiently managing bone metastasis.

The examination of cytokines and growth factors in bone remodeling and metastases presents valuable insights with inherent biases and strengths. An important asset lies in the comprehensive analysis of molecular mechanisms, including the interaction among different cytokines (such as IL-1, TNF) and growth factors (e.g., TGFβ), impacting osteoclast and osteoblast activity crucial for targeted therapy development [83]. These investigations often employ sophisticated genomic and proteomic tools to unravel intricate interactions, establishing a solid molecular comprehension supporting potential therapeutic strategies. Nevertheless, the research demonstrates biases, notably in extrapolating findings from animal models to human contexts. While animal studies offer fundamental preliminary insights, they may not consistently replicate human pathophysiology accurately, potentially leading to discrepancies in clinical relevance. Furthermore, the emphasis on specific cytokine pathways could overshadow other vital biological processes in bone metastasis, emphasizing the necessity for more comprehensive investigative strategies.

Additionally, studies frequently concentrate on biochemical facets without fully incorporating clinical outcomes or patient-reported symptoms, which are essential for assessing the practical efficacy of potential interventions [5]. This discrepancy highlights the importance of translational research that links laboratory discoveries with clinical trials to guarantee the transformation of theoretical advantages into tangible patient outcomes.

## 4. Role of Bone Turnover Biomarkers in Bone Metastases

### 4.1. Bone Turnover Markers

Bone turnover markers (BTMs) are released into the bloodstream as a result of an increase in osteogenesis or osteolysis. These markers are either enzymes, metabolites, or bone matrix proteins involved in the process of ongoing bone turnover [107]. Some of these markers may be proteins, ncRNAs, CTCs, ctDNA, or tissue-based markers. Their presence or abnormal expression reflects the nature of certain types of tumors. This can provide significant insight into the effects of bone metastases on bone turnover. They can be classified into bone formation or bone resorption markers that mirror increased bone deposition or bone breakdown, respectively. It is important to keep in mind that alterations in the levels of bone biomarkers are neither disease-specific nor location-specific. Rather, they provide information on bone metabolism in the whole body independent of an underlying cause. Regardless, the measurement and quantification of BTMs in the serum or urine of patients opens up possibilities for their utilization in the diagnosis, prognosis, and follow-up in skeletal metastases.

Biomarkers alone cannot be used to diagnose a tumor. They may be used as an adjunct to radiological imaging and clinical symptoms. They can also be used as a way to identify patients at high risk of developing bone metastasis or developing skeletal-related events [108]. This will enable targeted therapies for patients who will need it most while also mitigating their harm in patients who do not require such therapies. BTMs serve as a non-invasive and cost-effective method that has great clinical potential in bone metastasis identification and follow-up.

The use of BTMs is limited by preanalytic and biological sources of variability. Such factors, both modifiable (food intake, circadian rhythm) and nonmodifiable (age, gender), influence the levels of bone markers [109,110,111]. Moreover, the use of various cancer therapies affects the levels of bone markers [112]. This presents a major hurdle in the practical utility of these markers. Altered BTM levels in patients have to be interpreted in the context of the specific marker’s variabilities. Several approaches of reducing variabilities are being investigated. Knowing the sources of variability and the approaches employed to reduce them is of crucial importance for the interpretation of patient data. To reduce some of this variability, certain conditions are set for the collection and storage of a sample. Using automated standardized assays in accordance with international reference standards is recommended.

Our discourse concerning the clinical significance of bone turnover markers (BTMs) as tools for diagnosis and prognosis emphasizes their potential utility in the management of bone metastases. Nevertheless, delving further into this topic, we suggest that these markers also offer invaluable insights into the physiological reactions of bone-to-metastatic and therapeutic stresses. For example, monitoring changes in BTMs following treatment with anti-resorptive agents or other targeted therapies could facilitate more personalized adjustments to treatment strategies. Furthermore, with advancements in technology, the enhancement of more precise and sensitive assays for these markers may result in earlier identification and improved monitoring of disease progression. In our viewpoint, this has the potential to significantly alter existing paradigms towards a more dynamic and adaptable approach to treatment, wherein therapy is continuously tailored based on biomarker levels, thereby optimizing patient care and potentially enhancing outcomes in cases of bone metastatic disease.

### 4.2. Bone Formation Markers

#### 4.2.1. Bone Alkaline Phosphatase

Alkaline phosphatases are a group of homodimeric isoenzymes that catalyze the hydrolysis of phosphate monoesters by forming a phosphorylated serine intermediate at a basic pH (8–10) [113]. In humans, ALPs can be found in almost all body tissues. ALPs are categorized into tissue-specific (intestine, placenta, germ cell) and tissue-nonspecific (liver, bone, kidney) forms. The latter forms are clinically significant as the majority of ALPs found in the serum originates from the liver and bone in nearly equal amounts [114]. Tissue-nonspecific alkaline phosphatases are encoded by the ALPL gene on chromosome 1. The serum ALP level in healthy individuals displays variations in age and sex [115,116]. The bone isoform predominates during childhood and adolescence, while both liver and bone isoforms predominate in adulthood. Bone-specific alkaline phosphatase (BALP) is secreted by osteoblasts as a soluble homodimer [117]. BALP reflects bone mineralization as it provides inorganic phosphate for hydroxyapatite crystallization [118]. Hence, it can be utilized as a measure of increased bone formation and mineralization such as that seen in osteoblastic bone metastasis [107].

Alkaline phosphatases from different sources display different physiochemical properties [119]. Tissue-nonspecific ALPs (liver, bone, kidney) share an identical amino acid sequence; however, the carbohydrate and lipid side chains are distinct owing to differences in posttranslational modification. Multiple approaches have been devised to differentiate BALP from liver and intestinal isoforms. These methods include electrophoresis, manual or automated immunoassays, heat inactivation, selective chemical inhibition, high-performance liquid chromatography, and wheat germ lectin inactivation. Electrophoresis, on its own, fails to consistently distinguish the various isozymes due to the slight difference in the mobility between them. Combining electrophoresis on cellulose acetate with heat inactivation yields more dependable results [120]. BALP immunoassays utilize monoclonal antibodies that preferentially recognize the bone isoform. Immunoassays are rapid and reproducible, but cross-reactivity with the liver ALP has been observed (~20%) [121].

Several studies, including a meta-analysis, have found that BALP levels were significantly more elevated in patients with bone metastasis than in those without metastasis [122,123]. Other studies have highlighted the application of BALP in identifying patients at risk of developing SREs as elevated BALP levels correlated with their occurrence [124,125]. Another study suggested that elevated levels of BALP are associated with reduced disease-free survival in prostate cancer [126]. The results of a systematic review and meta-analysis that reviewed 25 studies demonstrated high BALP and ALP serum levels in bone metastatic breast cancer compared to in non-metastatic breast cancer patients. It also showed the pooled sensitivity and specificity of the ALP for breast cancer bone metastases were 0.62 and 0.86, and the area under the curve (AUC) was 0.80. The pooled sensitivity and specificity of BALP for breast cancer bone metastases were 0.66 and 0.92 and the AUC was 0.89. This further concludes that BALP and ALPs can bring about useful information for early detection of bone metastases in breast cancer patients [127].

#### 4.2.2. Osteocalcin

Osteocalcin is the most abundant non-collagenous protein in the bone extracellular matrix. In humans, it is encoded by the *BGLAP* gene on chromosome 1 [128]. As it is specifically produced by osteoblasts, it serves as a marker of bone formation. Its synthesis is under the influence of 1,25-dihydroxyvitamin D. Osteocalcin has three glutamate residues at positions 17, 21, and 24 that can undergo vitamin K-dependent post-translational carboxylation to form gamma-carboxy glutamic acid (gla). This carboxylation allows the calcium-binding Gla residues in osteocalcin to bind to the calcium ions in hydroxyapatite. In circulation, osteocalcin is rapidly degraded into fragments as it has a short half-life (~5 min). Osteocalcin can be measured as an intact molecule, large N-terminal mid fragments, and small C-terminal mid fragments [129]. These smaller osteocalcin fragments are thought to be released during the resorption of bone. Several factors affect the values of osteocalcin such as anticoagulants and high lipid amounts [130,131]. Osteocalcin assays may be based on manual enzyme-linked immunoassay (ELISA) or automated immunoassay analyzers.

In 1996, Ducy et al. created Ocn–/– mice that displayed higher bone mass and improved functional quality without impacting bone resorption [132]. This suggests that osteocalcin decreases the quantity of bone by decreasing bone formation. Recent studies contradict this evidence. The results of a study with osteocalcin-deficient mice showed collagen fibers to align normally but apatite crystallites to align randomly against collagen [133]. These results suggest that osteocalcin plays a role in bone quality and strength as opposed to quantity. Another recent study with triple-gene deletion for osteocalcin supports these findings [134]. It had been proposed that uncarboxylated osteocalcin acts as a hormone exerting influence on glucose metabolism, muscle mass, and testosterone synthesis [135]. Recent bodies of evidence have contradicted these findings [133,136]. More experiments are needed to further explore the functions of osteocalcin.

Studies on the utility of osteocalcin in the diagnosis and prediction of bone metastasis have been inconclusive. A study by Bayrak et al. found that osteocalcin levels were not significantly different in lung cancer patients with bone metastasis and in those with no metastasis [137]. Several studies showed that osteocalcin was decreased in NSCLC patients with bone metastasis [138,139]. In prostate cancer, some studies demonstrated significantly elevated osteocalcin levels in bone metastasis whereas other studies demonstrated no difference [140,141,142]. Taking into account its poor specificity, limited accuracy, and high biological variability, osteocalcin appears to be less than ideal for the diagnosis and prognosis of bone metastases.

#### 4.2.3. Type I Procollagen Propeptides: N-propeptide (PINP) and C-propeptide (PICP)

Procollagen 1 N-propeptide (PINP) and procollagen 1 C-propeptide (PICP) are proteins formed by the cleavage of type 1 procollagen. During collagen synthesis and following the formation of the triple helix, the amino- and carboxy-terminal ends of procollagen are enzymatically cleaved by endopeptidases releasing PINP and PICP, respectively [143]. Even though PINP reflects the synthesis of all type 1 collagen, it still acts as a sensitive indicator of bone formation as 70% of type 1 collagen is in bone tissue [143]. Moreover, most PINP is thought to be derived from bone owing to the much higher turnover rate than that in soft tissues.

PINP can be measured in the serum as an intact trimeric molecule, a dimeric molecule, a monomeric molecule, or fragments. However, PICP is released in a single molecular form. The clearance of PINP is dependent on the scavenger receptor and PICP on the mannose receptor in liver endothelial cells [144,145]. The monomeric form of PINP is cleared by the kidney [143]. Results of total PINP (trimeric and monomeric measurements) and intact PINP (trimeric measurements) are well correlated [146]. However, under several conditions such as chronic kidney disease, total PINP may be falsely elevated due to the decreased clearance of monomeric PINP [147]. The International Osteoporosis Foundation (IOF) and the International Federation of Clinical Chemistry and Laboratory Medicine (IFCC) recommend serum PINP as a reference marker for bone formation [148].

PINP, as well as PICP to a lesser degree, show potential utility to aid the diagnosis of bone metastases in breast and prostate cancer. Serum PINP was found to be significantly increased in patients with bone metastasis from breast cancer compared to in those with non-metastatic disease [149,150]. PINP was also elevated in bone metastatic prostate cancer with levels detectable 8 months before the first positive bone scintigraphy [151,152]. Similar findings were also reported in lung cancer primary [122]. PICP was shown to have a prognostic value in castration-resistant prostate cancer with skeletal metastases [153]. A prospective study in breast cancer with skeletal metastases showed that PINP was significantly (*p* < 0.05) elevated in patients with metastasis as opposed to in those without it. The use of PINP, BALP, and TRACP5b together aids in the early diagnosis of bone metastasis in postmenopausal women with luminal-type invasive ductal carcinoma [154].

### 4.3. Bone Resorption Markers

#### 4.3.1. Cross-Linked Telopeptides of Type I Collagen (CTX, NTX)

Type I collagen forms more than 90% of the organic bone matrix. Collagen has a triple-helical conformation with short nonhelical regions at both ends called telopeptides that contribute to the stabilization of fibrillar organization and tensile strength of collagen by forming intermolecular cross-links [155]. Pyridinium cross-links, pyridinoline [Pyr], or deoxypyridinoline [D-Pyr] are the intermolecular cross-linking compounds of collagen. The cross-links between collagen molecules and the C-terminal and N-terminal telopeptides of a different tropocollagen molecule occur at helix position 87 and 930, respectively [156]. During cathepsin K-mediated bone resorption, the cleavage of the telopeptides leads to the release of the N-terminal telopeptide (NTX) and C-terminal telopeptide (CTX) as degradation products of collagen. The N-terminal and C-terminal telopeptide fragments released into circulation are still connected to the collagen helix fragments by pyridinium cross-links. Hence, CTX and NTX can be used as a measure of bone resorption.

CTX and NTX are first released in an alpha configuration. Overtime, CTX undergoes isomerization and racemization, two post-translational modifications that result in the formation of four isomers: the native non-isomerized form (α-L) and three age-related forms that are an isomerized form (β-L), a racemized form (α-D), and an isomerized/racemized (β-D) form [157]. More precisely, the aspartic acid in position 19 of the C-terminal telopeptide of the α1-chain converts to the β form of aspartic acid [158]. As spontaneous β-isomerization occurs with protein aging, an increase in βCTX reflects mature aging bone while αCTX reflects newly formed bone. An increase in the ratio of α to β CTX indicates an increase in the turnover of new bone such as seen in malignant bone diseases, Paget’s disease, or even in physiological cases such as the normal growth of children [159,160]. Following their release into circulation, CTX and NTX are then cleared by the kidneys. While both serum and urine provide appropriate samples, CTX is most commonly measured in the serum and NTX in the urine for various reasons. Despite the availability of serum NTX assays, serum NTX values show less pronounced changes to antiresorptive therapy making urinary NTX (uNTX) a more preferred option [161]. The measurement of uNTX is usually expressed as a ratio to creatinine to account for urinary dilution. Serum CTX is preferred due to the large variability observed in urine CTX [162]. Due to the effects of circadian variation, reduced by a fasting state, on serum CTX, fasting morning samples are required [163]. The IOF and the IFCC have recommended serum CTX as a reference marker for bone resorption [148]. The C-terminal cross-linked telopeptide of type 1 collagen (ICTP), also known as CTX-MMP, is released following matrix metalloproteinase-derived bone degradation as opposed to cathepsin K [164].

Urinary NTX has shown a prognostic significance in patients with bone metastases receiving bisphosphonates. A pooled analysis of phase III trials of zoledronic acid showed that patients with high NTX levels had an increased risk in disease progression, skeletal complications, and death compared with those with low NTX levels (*p* < 0.001 for all) [165]. A normal NTX level was associated with a 52% lower risk of first pathological fracture (*p* < 0.0001) [166]. Additionally, the normalization of NTX, with bisphosphonate use, correlated with reduced risks of skeletal complications and death [167].

Various studies support its prognostic value in bone metastases from lung, prostate, and breast primary tumors [168,169]. A meta-analysis reviewing lung cancer patients with bone metastases showed that serum NTX has high clinical value in the diagnosis of metastases [170]. Serum NTX levels are significantly higher in patients with solid tumors with bone metastases than in those without them [171,172]. βCTX has displayed utility in the diagnosis of bone metastasis in lung and prostate cancers. A study conducted by Kong et al. on patients with lung cancer bone metastases demonstrated that they had significantly elevated βCTX and ICTP levels compared to those without metastases (*p* < 0.001 for both). ICTP displayed a higher sensitivity and accuracy than βCTX (75.4% vs. 65.6% and 72.9% vs. 68.8%, respectively) with a similar area under the receiver operating characteristic curve (0.85 vs. 0.83). Moreover, it significantly correlated with the extent of bone disease [173].

#### 4.3.2. Collagen Cross-Link Molecules (PYD, DPD)

Pyridinoline (PYD) and deoxy-pyridinoline (DPD), also known as hydroxylysylpyridinoline and lysylpyridinoline, respectively, are covalent pyridinium cross-links found in mature collagen. They contribute to the mechanical stabilization and rigidity of the collagen helix. Since these cross-links are formed during the extracellular maturation of collagen fibrils, their degradation reflects the degradation of mature collagen rather than newly synthesized collagen. While PYD can be found in both type 1 collagen in bone and type 2 collagen in cartilage, DPD is almost exclusively found in type 1 collagen in bone and dentin. Therefore, DPD is a more specific marker of bone resorption, despite it being less abundant [174]. PYD and DPD are renally eliminated without further metabolization. In urine, PYD and DPD are found in free (~40%) and peptide-bound (~60%) forms [175]. Moreover, dietary intake does not influence the excretion of pyridinium cross-links [176].

Measuring pyridinium cross-links can be done either by high-performance liquid chromatography (HPLC) or by ELISA [174]. Total pyridinium cross-links (sum of both free and peptide-bound forms) require acid hydrolysis of the sample to analyze the peptide-bound form [177]. While peptide-bound cross-links respond to bisphosphonate treatment, the free form has been shown to not respond as expected [178]. The free and total forms of DPD do not display comparable sensitivity in detecting short-term osteoclast inhibition [179].

A comparative study by Ebert et al. demonstrated that serum DPD and PYD levels were higher (*p* < 0.01) in lung cancer patients with bone metastases compared to in those with benign lung diseases and that the levels increased significantly with the number of metastases. The sensitivity and specificity for DPD were 83.7% and 34.5%, respectively, and for PYD they were 91.8% and 24.1%, respectively [122]. Another study showed that the urinary D-PYD level was statistically significantly higher in patients with lung cancer with bone metastases than in patients without bone metastasis (*p* < 0.05) [180]. D-PYD might be of use in diagnosing bone metastases.

#### 4.3.3. Tartrate-Resistant Acid Phosphatase

Tartrate-resistant acid phosphatase 5b (TRACP5b), a member of acid phosphatases, is an enzyme released by osteoclasts [181]. It hydrolyzes phosphate esters in an acid environment. TRACP 5b is thought to correlate to the number of osteoclasts [182,183]. It is initially synthesized as a proenzyme and is later activated [184]. TRAP is encoded by the *ACP5* gene [185]. Two isoforms exist in the serum: TRACP 5a, derived from macrophages and dendritic cells, and TRACP 5b, derived from osteoclasts [186]. TRACP 5b reflects the bone resorption rate and TRACP 5a does not [187]. Immunoassays can differentiate these two isoforms. They are antigenically identical but have minor biochemical differences. TRACP 5a contains a sialic acid whereas TRACP 5b does not [186]. They also have a different optimal pH with 5a being approximately 6 and 5b being 5 [188]. Recently, Mira-Pascual et al. developed a new TRAP sandwich ELISA that verified that in vitro-cultured CD14+-derived osteoclast secreted both TRACP 5a and 5b, suggesting that a considerable proportion of TRACP 5a originates from osteoclasts as well [189]. Following its release into circulation, it is metabolized in the liver and excreted by the kidney into urine.

In serum, TRACP 5b can be measured by enzymatic and immunoassay techniques. Enzymatic assays are nonspecific as serum levels of acid phosphatase are higher than in plasma due to the presence of many forms of TRACP derived from platelets and erythrocytes. High bilirubin levels interfere with serum TRACP 5b [190]. Renal function and food intake do not influence TRACP 5b and so it may have utility in measuring bone resorption in patients with renal impairment [191]. TRACP 5b activity correlates with baseline bone mineral density and with collagen markers of bone resorption [187,192].

Several studies have demonstrated that TRACP5b might be a useful marker for diagnosing bone metastases in breast cancer [149,154]. A prospective study of women with early-stage breast cancer with bone metastases showed that BALP, PINP, CTX, and TRACP 5b were significantly (*p* < 0.05) elevated in patients with metastasis as opposed to in those without. TRACP 5b was the most accurate marker with an AUCROC of 0.784 (95% CI: 0.651–0.916). The use of PINP, BALP, and TRACP5b together was the most accurate combination with an AUCROC of 0.889 (95% CI: 0.798–0.981) [154]. Studies show that serum TRACP 5b levels are significantly elevated in prostate and breast cancers with bone metastases as opposed to in those without metastases [140,193]. However, studies on lung cancer with bone metastases yielded inconclusive results [139,194,195]. Additionally, the levels of TRACP 5b respond rapidly and significantly to antiresorptive therapy and rise again with bone metastasis progression [186,196].

The discussion surrounding bone turnover markers (BTMs) in the context of bone metastases reveals both robust strengths and notable biases inherent in related studies. These markers, as indicators of bone resorption and formation, offer significant potential for the non-invasive management of skeletal metastases through diagnosis, monitoring, and prognosis.

BTMs provide a non-invasive means to monitor bone metabolism, offering insights that are valuable in managing bone metastases. Their application alongside imaging and clinical assessment can enhance the accuracy of diagnosing and tracking disease progression, thereby enabling timely and targeted therapeutic interventions. Elevated levels of specific markers like bone alkaline phosphatase (BALP) and C-terminal telopeptide (CTX) have been correlated with an increased risk of skeletal-related events and disease progression, highlighting their prognostic value. This association supports the use of BTMs in identifying high-risk patients who might benefit from early and aggressive treatment strategies. Utilizing BTMs can be cost-effective compared to more invasive or expensive diagnostic methods. This advantage is particularly significant in health systems where resources are limited, allowing for broader application and potentially better patient outcomes at a reduced cost.

With the above-mentioned strengths, we believe several biases exist in using BTMs. BTMs are subject to considerable biological variability influenced by factors such as age, gender, circadian rhythms, and diet. This variability can confound the interpretation of results unless appropriately controlled, potentially leading to misdiagnosis or inaccurate assessment of disease status.

Levels of BTMs can be affected by various cancer therapies, which may alter bone metabolism independently of the disease process itself. This interaction complicates the direct attribution of changes in BTM levels to metastatic activity alone, requiring careful consideration in clinical settings.

While BTMs provide valuable information on overall bone metabolism, they lack disease and location specificity. This limitation means that elevated BTM levels cannot conclusively indicate the presence of bone metastases without additional diagnostic information, as they could also reflect other bone-related conditions or systemic metabolic changes. The preanalytical phase, including sample collection and handling, and the analytical variability across different assay methods can introduce significant biases. Standardization of assay methods and adherence to strict protocols for sample handling are critical to minimize these effects and improve the reliability of BTM measurements.

While the use of BTMs offers several advantages in the context of managing bone metastases, the interpretation of these markers must be carefully managed considering their inherent biases and the broader clinical context. Advances in assay technology and better standardization of testing protocols could enhance the accuracy and clinical utility of BTMs, thereby solidifying their role in the personalized management of patients with skeletal metastases.

## 5. Genetic Mutations in Bone Metastases and Their Prognostic Significance

The search for genetic mutations associated with an increased propensity for primary cancers to metastasize to bone has identified several molecular and genetic targets. Genetic mutations vary based on the type and characteristics of the primary tumor. As such, the ability to metastasize to distant sites is not a uniform trait shared by all tumors, even from the same primary tumor. Cancer metastasis is not a spontaneous event. It is thought to be a sequential progression whereby tumor cells acquire mutations and respond to various signals in the microenvironment. It is important to note that the genetic profile of primary tumors can evolve with metastasis. Identifying genetic mutations allows for the prediction and tailored therapy of a patient’s bone metastasis. Currently, further studies and genomic profiling are needed to fully elucidate the genetic mutations that predispose to the propensity for bone metastasis.

### 5.1. EGFR

EGFR (epidermal growth factor receptor), a single-chain transmembrane glycoprotein, is one of four members of the ErbB family of tyrosine kinase receptors. These closely related receptors include the following: EGFR (also known as ErbB-1, HER1), HER2/neu (ErbB-2), HER3 (ErbB-3), and HER4 (ErbB-4) [197]. The binding of epidermal growth factor ligands to EGFR results in receptor activation and subsequent receptor tyrosine kinase autophosphorylation and multiple downstream signaling transduction. This leads to the activation of multiple pathways including the mitogen-activated protein kinase (RAS/MAPK) pathway and the phosphatidylinositol 3-kinase (PI3K/Akt) pathway [198]. The activation of EGFR contributes to cellular growth control mechanisms such as proliferation, differentiation, and survival of epithelial tissue.

EGFR is a driver of tumorigenesis. In contrast to normal cells, cancer cells often display overexpression of EGFR or amplifications of genes encoding growth factor receptors [199]. The intense signaling and pathway activation grants cancer cells the ability to proliferate, invade, and migrate, leading to cancer progression and metastasis [200]. Mutations in EGFR have been observed in a number of solid cancers including NSCLC, glioblastoma multiforme, colorectal, and breast cancers.

EGFR mutations are associated with an increased risk of bone metastasis and poor prognosis. A study by Krawczyk et al. demonstrated that activating mutations in the EGFR gene were significantly more frequent in lung adenocarcinoma bone metastases (75%) than in primary lung adenocarcinoma (12.8%, χ^2^  =  25.43, *p*  <  0.00001) or in adenocarcinoma metastasis to CNS (14.75%, χ^2^  =  15.09, *p*  <  0.0001) [201]. The high percentage of patients with lung adenocarcinoma bone metastasis suggests that EGFR mutation may promote metastasis formation. Another study conducted revealed that EGFR mutations were associated with a higher incidence of bone metastasis in lung adenocarcinoma. The median survival was 18.2 months following metastasis development and 21.8 months in patients without bone metastasis [202]. Other studies on lung cancer support these findings [203,204].

### 5.2. ESR1

Estrogen receptor 1 (ESR1) is a gene that encodes an estrogen receptor. Mutations in ESR1 are not usually detected in primary breast cancers; rather, they are found in estrogen-positive breast cancer patients who have been previously treated with aromatase inhibitor therapy. Hence, ESR1 mutations are thought to be selected by hormonal therapy [205]. This has been observed particularly in bone metastatic breast cancer, implying that the bone microenvironment might play a selective role [205]. Currently, there is insufficient evidence to establish the role of ESR1 mutations in bone metastasis. A study concluded that ESR1 mutation correlates with estrogen receptor expression, affecting about 14% of estrogen receptor-positive breast cancer bone metastases [206]. In an analysis of genes in breast cancer bone metastasis, the ESR1, GATA3, and MLPH genes were associated with an increased incidence of bone metastasis with an AUC of 0.702, 0.489, and 0.651, respectively. The AUC increased to 0.804 when the three genes were combined [207].

### 5.3. TWIST1

Twist-related protein 1 (TWIST1) is a dimeric basic helix-loop-helix (bHLH) transcription factor [208]. It is encoded by the TWIST1 gene on chromosome 7q21.2 and contains two exons and one intron [209]. The structure of bHLH proteins is characterized by a conserved domain containing a short stretch of basic amino acids followed by two amphipathic α-helices separated by an inter-helical loop [210]. The α-helices facilitate the interaction of TWIST1 with another bHLH transcription factor resulting in the formation of homo- or hetero-dimers. These dimers bind to the Nde1 E-box element (CATATG hexanucleotide sequences) resulting in the regulation of the expression of downstream target genes. TWIST1 plays an important role in organogenesis and embryological development, particularly mesoderm differentiation [210,211]. It also contributes to dorsal–ventral patterning during early embryogenesis [208]. Mutations in the TWIST1 gene can lead to Saethre–Chotzen syndrome, an autosomal dominant disease characterized by a wide range of malformations such as craniosynostosis (premature fusion of the skull), facial asymmetry, ptosis, and syndactyly (abnormal fusion of the fingers) [212,213]. Furthermore, TWIST1 is expressed in various invasive and metastatic cancers including breast, prostate, esophageal squamous cell carcinoma, and hepatocellular carcinoma, among others [214,215,216,217].

TWIST1 affects various biological processes that contribute to the initiation, progression, and metastasis of cancers [218]. The mechanisms through which it achieves cancer progression and invasiveness vary across different types of cancers. For instance, TWIST1 promotes epithelial–mesenchymal transition (EMT) which allows epithelial cells to transform into mesenchymal cells enhancing the ability of tumor cells to migrate from the primary tumor site to secondary tumor sites [218,219]. The expression of Twist1 in tumor cells is modulated by several regulators including signal transducer and activator of transcription 3 (STAT3), nuclear factor kappa B (NF-κB), steroid receptor co-activator 1 (SRC1), Msh homeobox protein (MSX2), and Hypoxia-inducible factor 1-alpha (HIF-1α) [220].

A study by Chang et al. showed that EGFR promotes bone metastasis in prostate cancer by down-regulating miR-1, a tumor suppressor, and activating TWIST1, an oncogenic gene. TWIST enhances the metastatic potential of prostate cancer and promotes cancer progression through epithelial–mesenchymal transition (EMT), the expression of dickkopf homolog 1 (DKK-1), and enhancing osteomimicry of prostate cancer cells [221]. In an analysis of 115 prostate cancer specimens by Yuen et al., higher TWIST expression in the primary cancer correlated with a higher risk of metastatic development (95% metastasized to bone) [222]. Increased EGFR expression seen in prostate cancer is associated with poor prognosis [223].

The aforementioned studies offer crucial insights into the genetic foundations of bone metastases in various types of cancer, with a specific focus on mutations present in EGFR, ESR1, and TWIST1. These discoveries play a fundamental role in elucidating the molecular pathways responsible for driving metastasis and in the creation of targeted treatment strategies. Nevertheless, it is important to address particular biases and strengths present in these research endeavors.

Identification of distinct mutations, such as those in EGFR observed in lung adenocarcinoma, and of their correlation with heightened bone metastasis presents precise targets for therapeutic measures and prognostic evaluations. This precision significantly enhances the clinical significance of the investigations.

The utilization of sophisticated genomic methodologies to profile mutations across diverse cancer types enables a comprehensive comprehension of the landscape of cancer metastasis. This inclusive approach has the potential to unveil shared pathways exploited by various cancers in their metastatic journey towards bone. Studies that establish connections between functional consequences, such as escalated metastatic capabilities linked to TWIST1 expression and its regulatory processes, contribute to a deeper insight into the biological mechanisms underlying metastasis. This knowledge is indispensable for the development of interventions aimed at disrupting these mechanisms.

Many of the studies conducted may exhibit selection biases owing to the retrospective nature inherent in genetic analyses. An illustration of this is seen in the study focusing on EGFR mutations, which specifically involves patients already presenting with bone metastases, potentially failing to accurately represent the broader cancer population. The impact of previous treatments, particularly evident in investigations of ESR1 mutations among breast cancer patients undergoing aromatase inhibitor therapy, has the potential to obscure results. Mutations that emerge as a result of treatment do not necessarily reflect the natural progression of the disease but rather indicate a response to the selective pressures imposed by the therapy. The presence of cancer heterogeneity, encompassing both intertumoral and intratumoral variations, can exert a significant influence on the outcomes of genetic studies. Discrepancies in the genetic profiles between primary tumors and their metastases may not be sufficiently addressed, thus leading to conclusions that lack universal applicability.

Although associations have been established between EGFR and TWIST1 mutations, unfavorable prognoses, and heightened metastatic susceptibility, the clinical relevance of targeting these mutations necessitates validation through prospective studies. The biological implications of these discoveries must be translated into clinical trials to ascertain their efficacy and safety in real-world settings.

On the whole, while these studies offer crucial insights into potential genetic targets for therapeutic intervention, it is imperative to interpret their findings in light of the methodological constraints they may possess. Improving the robustness of future research endeavors through prospective, multicenter trials, while also considering treatment histories and tumor heterogeneity, will be pivotal advancements.

In the examination of genetic mutations linked to bone metastases, a comprehensive account has been provided on the various genetic elements influencing the tendency of primary tumors to establish themselves in the bone microenvironment. Upon reflection of these discoveries, it is imperative to underscore not solely the diversity of these genetic modifications but also their potential as prognostic indicators and targets for therapeutic interventions. Each genetic anomaly, ranging from variations in EGFR to irregularities in ESR1, carries ramifications that transcend mere biological curiosity, presenting a framework for customized therapeutic strategies. The evaluation I have conducted proposes that the incorporation of thorough genomic profiling into standard clinical procedures has the potential to transform the treatment of bone metastases. Such an approach would enable the identification of individuals at heightened risk of bone metastasis and the utilization of personalized medical tactics geared towards intercepting the metastatic progression at an early stage. Additionally, the ongoing progress in CRISPR technology and other gene-editing mechanisms shows potential not only in comprehending but also in actively managing these genetic pathways to our therapeutic benefit. This proactive approach to genetic investigation could ultimately result in advancements in preventing or significantly delaying the onset of bone metastases in vulnerable persons, thereby restructuring the landscape of cancer therapy.

### 5.4. Other Genes

Other genes such as fibroblast growth factor receptor (FGFR), ataxia telangiectasia mutated (ATM), cyclin-dependent kinase 12 (CDK12), hepatocyte nuclear factor 1 alpha (HNF1A), adenomatous polyposis coli (APC), and tumor protein 53 (TP53) have also been implicated in lung cancer bone metastasis [224]. In a study on the patterns of metastasis in breast cancer, BRCA2 mutation carriers frequently developed bone metastasis (75%) compared to noncarriers (53%) and BRCA1 mutation carriers (37%) [182]. Moreover, estrogen-positive breast cancer metastasizes to bone more frequently than estrogen-negative breast cancer [225].

## 6. Bone Metastasis: Clinical Features

### 6.1. Bone Pain

Bone pain, which affects 60–84% of patients with advanced cancer due to bone metastases, largely reduces quality of life and functional status [226,227]. Patients describe this pain as moderate to severe, dull, and worsening over time, exacerbated by tumor growth and bone remodeling, leading to incident pain despite ongoing analgesia. The pathophysiology involves complex interactions among tumor, bone, inflammatory, and nerve cells, causing neuropathic and nociceptive pain, with inflammatory and mechanical pain arising from cytokine release, periosteal irritation, and bone pressure or mass effect [228,229]. Osteoclasts, key players in bone resorption, are associated with cancer-induced bone loss and pain, implying that the acidic environment and activation of the acid-sensitive nociceptors participate in pain perception [227].

Epidemiology of bone metastases shows that the most common ones are associated with breast, prostate, and lung cancer; moreover, metastases in the vertebrae, pelvis, and long bones are the most frequent [226]. The extent of pain does not always correlate with the size of bone lesions, hence the need for unique pain-relieving strategies [226]. Both tumor and stromal cells contribute to the sensation of pain by secreting different factors that sensitize or stimulate primary afferent neurons, including NGF (nerve growth factor), which is fundamental in the genesis of bone cancer pain because of its effects on sensory neurons [227]. Inflammatory cytokines and chemokines, such as IL-1β and TNF-α, contribute to pain induction [230]. Other factors like ATP, TGF-β1, IGF-1, and Sclerostin are implicated in CIBP, suggesting potential targets for intervention.

### 6.2. Hypercalcemia

Hypercalcemia in cancer results from parathyroid hormone-related protein (PTHrP) secretion or activation of the receptor for nuclear factor-kB ligand, with bone metastases and increased osteoclastic activity accounting for about 20% of instances [231,232]. Being common in up to 44.1% of cancer patients, especially in advanced stages, hypercalcemia can lead to severe metabolic issues, significantly impacting morbidity and mortality—about 50% of affected patients die within a month [233].

Osteoclasts, rather than tumor cells, drive bone degradation in osteolytic metastases, with tumors boosting osteoclast formation and function, thus enhancing bone resorption and hypercalcemia [234]. Hypercalcemia, often symptomless, can manifest in neuropsychiatric, gastrointestinal, renal, and cardiovascular symptoms and exacerbate pain in cancer sufferers [235,236].

### 6.3. Pathological Fractures

Pathologic fractures impact 10–30% of cancer patients, often targeting long bones’ proximal regions, predominantly the femur in over 50% of cases [229]. Rib fractures and vertebral collapses also occur, leading to kyphoscoliosis and restrictive lung disease, with breast cancer accounting for 60% of these fractures and lung cancer 10%.

Lytic lesions cause fractures in weight-bearing bones, compromising both cortical and trabecular structures. In patients with bone metastases or a cancer history, sudden pain onset necessitates immediate pathologic fracture assessment, particularly if pain is the only symptom in the upper extremity and spine [237].

Predictors of fractures include large lytic lesions with cortical erosion [231]. Mirels’ scoring system, based on lesion site, nature, size, and symptoms, suggests surgery for scores > 7 and indicates over 50% fracture risk for scores ≥ 10. Advanced tools, including computed tomography, are being explored for assessing high-risk fracture sites. Pathological fractures also contribute to cancer-induced bone pain (CIBP).

### 6.4. Neurological Symptoms

Approximately 70% of cancer patients have metastatic disease at death, with up to 40% involving the spine [238]. Spinal cord compression affects 5–10% of these individuals, rising to 40% among those with non-spinal bone metastases. This condition often results in radicular/neuropathic pain, characterized by stabbing, shooting, or burning sensations following a dermatomal pattern [239].

Vertebral metastasis, facilitated by rich vascular and lymphatic networks, can compress the spinal cord, constituting a severe oncological emergency. This compression, whether from metastasis or vertebral extension, risks neurological deficits, ranging from pain, paresthesia, and motor weakness to incontinence, loss of sphincter control, and paraplegia [240]. Typically, pain precedes malignant spinal cord compression (MSCC) diagnosis by around two months, highlighting a slow recognition process due to symptom subtlety. Late-stage symptoms include urinary retention and impotence, while lesions on the conus medullaris may prompt early autonomic issues affecting bladder, rectum, and genitalia [231].

## 7. Diagnosis of Bone Metastases

Diagnosing bone metastases, a condition where cancer cells spread from their original site to the bones, involves a multifaceted approach combining advanced imaging techniques, laboratory tests, and biopsies. This complex process aims to accurately identify the presence of metastatic disease, assess its extent, and guide treatment planning. The following paragraphs outline the key diagnostic tools used in this process, including the roles of radiographs, CT scans, MRI, PET scans, and the importance of laboratory tests and biopsies in confirming the diagnosis (Table 1).

### 7.1. Radiography

Radiographic imaging, despite its utility in screening for bone metastases, often reveals its limitations in sensitivity, detecting only 44 to 50 percent of cases. This method can identify the nature of bone lesions—whether lytic or osteoblastic—and fracture lines, yet it struggles with early detection of metastases. In comparison, CT scans elevate the diagnostic process with 74% sensitivity, offering detailed insights into bone architecture essential for treatment planning and identifying metastases earlier than radiographs [241,242]. They are particularly valuable in scenarios where MRI is not viable, providing clarity on fractures and spinal cord compressions. However, CT scans cannot rival the soft-tissue contrast achieved by MRI.

### 7.2. MRI and PET Scans

MRI technology stands out for its exceptional sensitivity and specificity, making it indispensable in diagnosing bone metastasis. Its ability to provide detailed images of bone lesions, soft tissues, and solid organs, along with its superior performance in spinal metastasis detection, set it apart [243]. Although susceptible to movement and metallic artifacts, MRI’s comprehensive view supports not only diagnosis but also the monitoring of treatment responses. PET scans, employing FDG-PET and integrated FDG-PET/CT, further complement the diagnostic arsenal by offering metabolic imaging capable of detecting distant metastases, significantly aiding in the clinical staging and restaging of various cancers [244].

Whole-body scintigraphy is an important diagnostic technique for identifying bone metastases, especially when employing technologies such as bone scintigraphy and MRI. Studies indicate that whole-body MRI is often more sensitive and specific in detecting bone metastases compared to bone scintigraphy. However, bone scintigraphy continues to be widely utilized due to its widespread availability and cost-effectiveness. For instance, a comparative analysis showed that whole-body MRI was notably superior to bone scintigraphy in detecting bone metastases in patients with established malignant tumors, emphasizing the higher diagnostic accuracy and improved interobserver agreement of MRI [245]. A separate study has verified the heightened sensitivity of MRI in comparison to that of scintigraphy for patients with kidney cancer. Additionally, it highlighted MRI’s capability to identify soft-tissue illnesses, a task that bone scintigraphy is unable to perform [246].

Although MRI provides superior diagnostic accuracy for bone metastases, bone scintigraphy remains essential in clinical settings, particularly in cases where MRI accessibility or expenses are limiting constraints. Both approaches are crucial for a thorough evaluation of metastatic dissemination in cancer patients.

### 7.3. Other Diagnostic Techniques

In addition to imaging techniques, the diagnosis of bone metastasis incorporates laboratory tests to measure serum alkaline phosphatase levels, a marker of osteoblastic activity commonly elevated in prostate and breast cancer cases. Tumor markers and bone biopsies remain fundamental, with needle biopsies and liquid biopsies providing critical insights into the cancer’s type and origin [247,248]. While imaging technologies continue to advance, integrating AI for better precision and efficiency, these diagnostic tools collectively enhance the accuracy of detecting bone metastases, ultimately improving patient care through more informed treatment decisions and monitoring.

### 7.4. Effectiveness and Limitations of Different Diagnostic Tools

The array of diagnostic modalities used to identify bone metastases varies in efficacy and suitability, each exhibiting distinct strengths and limitations. Radiography, although widely utilized and valuable for screening purposes, demonstrates limitations in its sensitivity, detecting just 44–50% of metastatic instances. This conventional approach is frequently surpassed by CT scans, which present a sensitivity of 74% and offer more intricate bone structure images, which are essential for prompt identification and treatment strategizing.

Conversely, MRI emerges as a frontrunner in the domain owing to its heightened sensitivity and specificity. It excels in visualizing both bone lesions and the adjacent soft tissues, proving to be particularly adept at identifying spinal metastases. Its primary drawback lies in its susceptibility to motion and metallic artifacts that may compromise image fidelity.

PET scans introduce an additional layer of diagnostic accuracy, utilizing metabolic imaging to identify cancer dissemination and significantly aiding in clinical staging. Whole-body MRI and bone scintigraphy are commonly contrasted for their effectiveness. Although MRI delivers superior diagnostic precision and sensitivity, bone scintigraphy remains preferred in numerous clinical contexts due to its cost efficiency and wider availability.

Laboratory analyses and biopsies complement these imaging modalities by confirming the presence of metastases and aiding in determining the cancer’s point of origin, thereby fortifying and enhancing the entire diagnostic procedure.

## 8. Management of Bone Metastases

Current treatments for neoplasm aim to decrease the size of the tumor and block the process of cell growth, which subsequently cause the destruction of the bones, leading to fractures and pain [249]. Their sphere of activity is mostly limited to the symptomatic control of the patients, now usually called palliative care, rather than curative treatment [249]. Many other management protocols are available based on patient conditions (Table 2).

### 8.1. Bone Pain Control Techniques

Pain management in bone metastasis follows the WHO’s “Three Steps Analgesic Ladder” whereby non-opioids are the starting points while opioids are the latter steps as may be needed [239]. Non-steroidal anti-inflammatory drugs (NSAIDs) which can be administered together with opioids have shown their short-term effectiveness in managing cancer pain [250]. Anticonvulsants and tricyclic antidepressants historically used for neuropathic pain could be the ones prescribed [250]. The intensive use of opioids for managing extreme pain is crucial; however, care must be taken so as to give adequate dosage. However, it is questionable how well weak opioids prepare for the pain and here strong opioids like buprenorphine and oxycodone are recommended [250].

### 8.2. Strengthening Bone Structure

Although targeting bone metastases is considered the most troublesome of all bone complications, bisphosphates as powerful inhibitors of bone resorption help to hinder the disease progression through a reduction of the most common complications [251]. They also provide to a limited extent a compressive Wada spinal cord reaction and may have associated, adverse effects [252]. Clinical trials have demonstrated that denosumab has additional competence compared to bisphosphonates due to its capacity to suppress bone-related complications and pain without affecting bone resorption or osteoclast formation [250]. Despite the progress, healthcare providers concentrate their treatment efforts on these two drugs as part of the scope of bone metastasis management [253]. A recent randomized phase 2 trial evaluated the effectiveness and safety of different dosing intervals of zoledronic acid (ZA) in patients with lung cancer who have bone metastases. It compared the effects of administering 4 mg of ZA every four weeks versus every eight weeks on the incidence of skeletal-related events (SREs). The study, conducted at eight Japanese hospitals, involved 109 patients and found no significant difference in the time to the first SRE, SRE rates at one year, or any secondary outcomes like pain, toxicity, and overall survival between the two dosing schedules. This indicates that extending the dosing interval to eight weeks may be as effective as the more frequent four-week schedule without compromising patient outcomes [254].

A recent study examined the impact of denosumab on disseminated tumor cells (DTCs) in patients with breast cancer undergoing neoadjuvant chemotherapy (NACT). The study found that denosumab, when added to nab-paclitaxel-based NACT, did not significantly increase the eradication rate of DTCs compared to NACT alone. Initially, 25.7% of patients exhibited DTCs at baseline, and there was no significant predictive value of DTC presence for response to NACT. Post-treatment, DTCs were eradicated in 77.8% of the NACT plus denosumab group and 69.6% of the NACT alone group, showing no significant difference. This suggests that short-term use of denosumab does not enhance DTC eradication in the context of neoadjuvant treatment for breast cancer [255].

### 8.3. Efficacy of Hormone Therapy

In breast and prostate cancers, hormone therapy is key in the management of bone metastatic growth. Therapies like bilateral oophorectomy and tamoxifen as antiestrogens have given promising results and continue to fight breast cancer [256]. As for prostate cancer in the first place, hormones therapy is an efficient way to reduce pain and thus improve the health of patients.

### 8.4. Radiation Therapy Application and Radiopharmaceutical Usage

Both radiotherapy and painkillers have a palliative treatment effect, which is more efficient in terms of relief from the pain and bone regrowth progress compared to the other categories. One can easily understand the kind of importance of this technique in the condition when patients’ bone pain, which is not receptive to commonly used pain medications, is a concern. For example, agents such as strontium-89 and radium-223 are applicable to metastatic bone pain disorders while multi-metastatic affected sites target the treatment. Radium-223 mentioned here generates the dose directly to the bone metastases and it prolongs the life of the patient and reduces pain through inhibition of the proliferation of the cancer cell. When cancer metastasis is widespread, radiopharmaceuticals administration, in combination with that of others like bisphosphonate treatments, enhances therapy results [239]. Radiofrequency ablation (RFA) substitutes for the traditional method of bone metastasis pain attains a huge rate of accuracy [250]. With the help of RFA or radiofrequency ablation, it is possible to introduce high-frequency currents to get rid of abnormal tissue, which, in turn, can reduce pain and tumor size [239]. However, on the downside, the possible nerve damage cannot be overlooked, but the speedy pain relief makes RFA generally safe [252]. External beam radiation therapy, also known as EBRT, is generally considered the gold standard of care and is among the most often employed methods for the treatment of patients who are experiencing pain as a result of bone metastases. Despite the fact that, in more than fifty percent of patients, pain reduction is experienced within one to two weeks following EBRT, at least 40% of treated patients do not achieve pain relief [257,258]. A phase II clinical trial compared the effectiveness of MRI-guided focused ultrasound surgery (MRgFUS) and external beam radiation therapy (EBRT) in treating pain caused by bone metastases. The trial included a total of 198 patients, with 100 of them receiving MRgFUS and the remaining 98 undergoing EBRT. The results showed that MRgFUS had a substantially greater percentage of pain response at the 1-month follow-up (91%) compared to that of EBRT (67%). At the 12-month follow-up, MRgFUS showed superior effectiveness with a response rate of 92%, compared to EBRT’s response rate of 61%. Furthermore, MRgFUS had better results in evaluating the quality of life, including assessments of physical function, hunger, and nausea, with a lower occurrence of negative events (15%) compared to that of EBRT (24%). The findings indicate that MRgFUS is a feasible and efficient substitute for alleviating pain in individuals with bone metastases. It presents fewer adverse effects and enhances the overall quality of life when compared to traditional radiation therapy [259].

### 8.5. Chemotherapy’s Role

It has been found that response to chemotherapy is different for individual cases in terms of the type of tumor and the chemosensitivity of the patient [252]. Electrochemotherapy, a therapeutic illusion which accompanies electrical pulses and chemotherapy drugs, is the new hope for resistant bone metastasis [260]. Apart from its complementation role, it can be used for communication with doctors about treatments like radiotherapy which will lead to a holistic approach for bone metastasis management [261].

### 8.6. Surgical Interventions

Surgery attempts to relieve pain; return functioning; and, in the case of severe nerve compression, prolong a patient’s life. A range of procedures go from minor to highly elaborate. They can be determined by a patient’s health and a tumor’s parameters. Invasive methods, as radiofrequency ablation, and percutaneous vertebroplasty give a chance for patients to avoid spine surgery. A study by Xu et al. evaluates the effectiveness of combining percutaneous vertebroplasty (PVP) with ^125^I seed implantation for treating lumbosacral vertebral osteoblastic metastases. The results indicated significant improvements in pain management and physical condition for patients receiving the combined treatment compared to those receiving only PVP. Specifically, pain levels significantly decreased (*p* = 0.000) and Karnofsky Performance Status scores improved in the combination group. Survival analysis showed that the combination therapy group had a significantly better survival rate compared to that of the PVP-only group (*p* = 0.038). Moreover, factors such as age, the extent of spinal metastasis, and the primary tumor’s growth rate were identified as independent predictors of long-term survival. This suggests that the integration of ^125^I seed implantation with PVP could potentially enhance clinical outcomes in patients with this form of metastatic cancer [262].

### 8.7. Advancements in Targeted Therapies

Consequently, identification of unique molecular mechanisms, for instance TGFβ signaling, opens new approaches that could end the spread of bone metastases [263]. An example of the cure would be monoclonal antibodies and antisense oligonucleotides which interfere with the activity of TGFβ, demonstrating the possible therapy in cancer treatment and osteoporosis prevention of bone resorption [264]. Studies are going on whether such highly specialized medicine is effective when it is utilized in a real-life setting [265].

## 9. Conclusions and Future Direction

Throughout this review, we have synthesized the complex interactions that underpin bone metastasis, noting both the progress and the gaps in our current understanding. From an editorial perspective, I advocate for a future where these gaps are addressed not just through incremental advances but through transformative research approaches. Integrating emerging technologies such as artificial intelligence and advanced computational models to analyze large datasets could redefine our predictive capabilities and enhance the personalization of therapy for bone metastasis. Moreover, coupling genetic profiling with the development of new targeted therapies could lead to highly personalized medicine that is tailored to the unique genetic and molecular landscape of each patient’s tumor. This approach would not only refine therapeutic strategies but could significantly prolong survival and improve quality of life for patients suffering from bone metastases, marking a new era in the management of this challenging condition.

## Figures and Tables

**Table 1:** This table provides a comprehensive overview of the diagnostic methods for bone metastases, including the main applications, advantages, and limitations of each tool.

Diagnostic Tool	Description	Applications	Advantages	Limitations	References
**Radiography**	Basic imaging technique using X-rays.	Initial screening for bone lesions	Widely available, fast, and cost-effective	Less sensitive for early metastasis detection	Section 7.1
**Computed tomography (CT)**	X-ray-based technique that provides cross-sectional images.	Detailed bone architecture, assessment of fractures	High resolution, good for assessing complex anatomy	Limited in soft-tissue contrast	Section 7.1
**Magnetic Resonance Imaging (MRI)**	Uses magnetic fields and radio waves to produce detailed images.	Spinal metastases, soft-tissue detail	High sensitivity and specificity, excellent soft-tissue contrast	Can be expensive, not always available	Section 7.2
**Positron Emission Tomography (PET)**	Imaging that uses radioactive substances to show activity.	Functional imaging of metabolic activity	Highly sensitive for detecting active disease	High cost, limited availability	Section 7.2
**Bone scintigraphy**	Uses radiotracers to detect bone changes.	Whole-body screening for skeletal metastasis	Sensitive for detecting bone remodeling	Less specific, cannot differentiate benign from malignant lesions	Section 7.2
**Biopsy**	Removal of tissue samples for microscopic examination.	Confirming diagnosis of suspicious lesions	Definitive diagnosis of metastasis	Invasive, potential complications	Section 7.2

**Table 2:** This table offers a detailed overview of each management strategy for bone metastases, including their specific clinical indications, benefits, and potential side effects.

Management Strategy	Description	Indications	Benefits	Potential Side Effects	References
**Bisphosphonates**	Drugs that inhibit osteoclast-mediated bone resorption to reduce skeletal-related events.	Bone pain, prevention of fractures	Reduces bone pain and risk of fractures	Kidney dysfunction, osteonecrosis of the jaw	Section 8.1 and Section 8.2
**Denosumab**	A monoclonal antibody that inhibits RANKL, preventing osteoclast formation and function.	Similar to bisphosphonates	Similar to bisphosphonates, but with potentially longer duration of action	Hypocalcemia, osteonecrosis of the jaw	Section 8.2
**Radiopharmaceuticals**	Radioactive substances used to target and kill cancerous cells in the bone.	Metastatic bone pain resistant to therapy	Directly targets bone lesions, reducing pain	Bone marrow suppression, radiation sickness	Section 8.4
**External beam radiation**	Radiation therapy aimed at specific areas to manage pain and prevent fractures.	Localized bone pain, risk of fractures	Effective pain relief, can prevent spinal cord compression	Skin irritation, fatigue	Section 8.4
**Surgical intervention**	Procedures like vertebroplasty, kyphoplasty, and stabilization surgeries.	Structural compromise, severe pain	Immediate pain relief, stabilization of bone	Surgical risks, infection	Section 8.6
**Chemotherapy and hormonal therapy**	Systemic treatments based on the primary cancer type, targeting metastatic cells.	Widespread metastatic disease	Can reduce tumor burden, manage systemic disease	Varies by agent: nausea, hair loss, etc.	Section 8.3 and Section 8.5
**Targeted therapy**	Drugs targeting specific molecular pathways involved in cancer growth, like tyrosine kinase inhibitors.	Hormone-sensitive cancers	Can specifically target and inhibit cancer growth	Varies by agent: hypertension, rash, etc.	Section 8.7

## Data Availability

Data sharing is not applicable.
